# Learning Disabilities: Reducing CTPLD West Psychiatry Clinic DNAs by 20% by Offering a More Person-Centred Service

**DOI:** 10.1192/bjo.2024.390

**Published:** 2024-08-01

**Authors:** Kerenza MacDaid, Labib Hussain, Sarah Maber

**Affiliations:** 1 Surrey and Borders Partnership NHS Trust, Guildford, United Kingdom; 2 Surrey and Border Partnership NHS Trust, Guildford, United Kingdom; 3 Surrey and Borders Partnership NHS Trust, Chertsey, United Kingdom

## Abstract

**Aims:**

To explore reasons why patients under the learning disability community psychiatry team were not attending their appointments.

To explore patient preferences for type of appointments offered (F2F, telephone or video) aiming to improve access and attendance, whilst promoting reasonable adjustments for patients with learning disabilities.

To improve efficiency in terms of number of patients seen and reduce wasted clinical and admin time in relation to DNA appointments, by reducing the ‘did not attend’ (DNA) rates by 20%.

**Methods:**

With the support of the QI team using methodology including fishbone diagrams and PDSA cycles, electronic data on the number of DNA psychiatry appointments were collated from January 2022 onwards and recorded monthly.

Each DNA appointment was coded with a reason given for non-attendance. The data was then reviewed and analysed to identify any common themes around non-attendance.

A questionnaire was initially trialled with a small sample of patients who had not attended to explore reasons for non-attendance and preference regarding appointment type. The questionnaire was then sent to all CTPLDW patients who had missed appointments. An easy-read version was also sent to promote accessibility amongst the patient group. The data was collated and reviewed.

**Results:**

Most common reasons for patients not attending their psychiatry outpatient clinic appointments under the CTPLDW team were identified:

2022: 35 patients DNA – 28.6% citing communication/correspondence issues.

2023: 30 patients DNA – 33.3% citing communication/correspondence issues.

Additional reasons for non-attendance included issues with residential homes, sickness and transport.

**Conclusion:**

An anecdotally high number of DNAs were noted by CTPLDW. The data collected thus far has helped us to define and understand the issues. The main factors identified revolve around communication and correspondence of appointment times.

The next step in our quality improvement project is to trial text reminders for patients and carers to assist in remembering appointments, to assess whether this change idea helps to decrease the number of DNAs.

Future change ideas include development of resources to support attendance (e.g. adjusted appointment letters with QR codes for access/maps, reminder letters in easy-read format and video tours).

CTPLDW would like to offer a more personalised approach with a service that promotes reasonable adjustments and reduces barriers to access, thereby reducing the number of DNA appointments.

